# Application of Herbal Medicines with Bitter Flavor and Cold Property on Treating Diabetes Mellitus

**DOI:** 10.1155/2015/529491

**Published:** 2015-10-18

**Authors:** Hongdong Chen, Jing Guo, Bing Pang, Linhua Zhao, Xiaolin Tong

**Affiliations:** Guang'anmen Hospital, China Academy of Chinese Medical Sciences, Beijing 100053, China

## Abstract

Diabetes mellitus has been a global pandemic. Traditional Chinese Medicine has been used on diabetes mellitus for thousands of years and the modern Chinese medicine studies have found a curative effect of herbal medicine with bitter flavor and cold property on diabetes. This review will introduce the theory summary of flavor and property in TCM, argument basis, the evidences from clinical trails and animal experiments, the possible antidiabetic mechanisms, and advantages on lowering glucose of herbal medicines with bitter flavor and cold property and take rhizome, Chinese rhubarb, and *Momordica charantia*, the three herbal medicines with bitter flavor and cold property, as examples to illustrate the exact antidiabetic effect. It is hoped that this review can provide some ideas and inspiration for the treatment of diabetes with herbal medicine.

## 1. Introduction

Diabetes mellitus (DM), as a noncommunicable chronic disease, has become the leading cause of mortality and disease burden worldwide. The prevalence of diabetes has increased significantly in recent decades and is now reaching epidemic proportions in China. According to the research, in 2013, the estimated prevalence of diabetes among a representative sample of Chinese adults was 11.6%, about 113.9 million people, and the prevalence of prediabetes was 50.1%, about 493.4 million people [1]. Faced with this situation, China has been throwing manpower, material resource, and money to study DM. Traditional Chinese Medicine (TCM) as a treatment of DM has made great progress in recent years and the effects have been recognized. In the formulas that have been demonstrated to be effective with DM, we find that herbal medicines with bitter flavor and cold property play an important role in the treatment. Rhizoma Coptidis,* Rheum officinale*,* Momordica charantia*,* Rhizoma Anemarrhenae*, and* Ramulus Euonymi*. Here we describe these representative herbs and their application in diabetes as well as proposing mechanisms of action and efficacy for treating diabetes in order to make the Western people know more about antidiabetic TCM.

## 2. The Theory Summary of Bitter Flavor and Cold Property

The theory of four properties and five flavors is the special attribute of herbal medicines (HMs) and a part of the theory of drug properties. HMs have four properties, cold, hot, warm, and cool, and five flavors, sour, bitter, sweet, pungent, and salty. Different property and flavor have different peculiarity. With cold-hot mixed, Yin and Yang blended, the theory guides the clinical medication for TCM doctors. (Yin and Yang are two opposite aspects that run through everything. In general, Yang represents that which is light or clear, functional, hyperfunctional, dynamic, ascending, or hot, while yin represents that which is heavy or cloudy, substantial, hypofunctional, static, descending, or cold.) “Su Wen” (*Plain Question, title of a traditional Chinese medical book, a component of Yellow Emperor's Classic of Internal Medicine*) refers to “sweet and pungent flavors inducing perspiration and pertaining to Yang, sour and bitter flavors which can cause emesis and purgation being attributive to Yin.” So sweet flavor belongs to Yang and bitter belongs to Yin. Yin and Yang restrict each other to be in equilibrium; then, the body works healthy. Bitter flavor can consolidate body, ease dampness, and purge heat. Consolidating body is the main action of bitter flavor, consisting of consolidating Qi (*vital energy*), consolidating Yin, and consolidating the kidney [2]. The basic effect of cold property is to treat disease with pathogenic heat, namely, “treating heat with cold drug.” Cold property is the common property of HMs that can release or remove heat syndromes. Bitter flavor and cold property both belong to Yin, with bitter flavor discharging heat and cold property treating heat syndromes. Because of that similarity, HMs with bitter flavor are mostly with cold property [3–5]. Bitter flavor and cold property were first called together in “Su Wen·Zhi zhen yao da lun” that recorded the treatment direction of HMs with bitter flavor and cold property and proposed that bitter flavor and cold property could clear heat and dispel dampness. In “Bei ji qian jin yao fang” (*a book name: Prescriptions Worth a Ten Thousand Gold For Emergencies*), it was emphasized that HMs with bitter flavor and cold property had the effect of draining fire. They had the effect of discharging repletion heat in qi-aspect and exuberant evil qi as well as the effect of enriching and supplementing. To sum up, bitter flavor and cold property are common functions of a class of HMs that can not only release or remove heat syndromes but also clear and discharge heat, dry dampness, and consolidate Yin [6].

## 3. The Argument Basis of HMs with Bitter Flavor and Cold Property on DM: The Core Pathogenesis of the Early and Middle Stages of DM Is Center Fullness and Internal Heat

In TCM, DM belongs to the category of Xiaoke disease whose core pathogenesis is “Yin deficiency and dryness-heat” diagnosed based on “three excessive and one loss” symptoms, excessive fluid drinking, excessive food consumption, excessive urination, and weight loss. Compared with prior living environment, the modern diet has changed significantly resulting in the changing of main diabetic population from marasmus population with “three excessive and one loss” symptoms to obesity group. 90% of DM patients are T2DM, and 85% of T2DM patients are obese [7]. An analysis of the patient's body type survey shows approximately 72.7% of the diabetic patients are obese or overweight and among them 79.7% are abdominal obesity, affirming that abdominal obesity population is the main diabetic group [8].

The typical “three excessive and one loss” symptoms are usually occurring at the late stage of DM, so most diabetic patients have no expression of the symptoms. The traditional TCM theory of Yin deficiency and dryness-heat makes it more difficult to obtain a satisfactory curative effect during the treatment of diabetes; therefore, novel theories have been proposed [9]. Researchers, having studied the pathogenesis of diabetes mellitus in recent years, put forward that the early and the middle stages of DM belong to “Pi Dan” (*an ancient disease name: the spleen-warm syndrome*) category whose initiating agents are overeating and less-sporting and the core pathogenesis is center fullness and internal heat [10–12]. Overeating fat and sweet food inspire the function of spleen and stomach, leading to dyspeptic retention in center burner and transform into heat with the passing of time. This mainly implicates spleen, stomach, intestine, and liver-gallbladder bringing about stomach heat, intestinal heat, gastrointestinal heat or liver-gallbladder heat [13, 14]. So in the early and middle stages of DM, the method of treatment is clearing heat and draining fire what HMs with bitter flavor and cold property are exactly good at.

## 4. The Evidence of HMs with Bitter Flavor and Cold Property in the Diabetic Treatments

### 4.1. The Evidence of TCM Formulas Constituted with HMs with Bitter Flavor and Cold Property in the Diabetic Treatments

In China, randomized controlled clinical trials were conducted for DM with compounds of TCM which are constituted with HMs with bitter flavor and cold property.

Lian et al. applied Kaiyu Qingre Fang (including Scutellaria, Rhizoma Coptidis, and Chinese rhubarb) to treat DM, reducing the glycosylated hemoglobin by 1.32% [15]. This effect was attributed to regulation of glucose by improving late phase *β*-cell secretion of insulin. This could also contribute to decreased insulin resistance [16]. What is more, the formula was documented to reduce fatty acid synthase activity by modifying overexpression of sterol regulatory element binding protein-1c to modulate glucose and lipids in diabetic patients [17, 18].

Tong's group reported that TM81 (which includes Rhizoma Coptidis, Chinese rhubarb, and radices trichosanthis) as the diabetic treatment can reduce glycosylated hemoglobin by 1.18% [19]. They postulated that TM81 triggered insulin secretion in a high-glucose environment and that this compound was similar to rosiglitazone's ability to improve insulin secretion by islet cells by significantly increasing PPAR*α* [20]. Lian et al. reported that Qingre Jiangzhuo Fang (Chinese rhubarb,* Momordica charantia*,* Anemarrhena*, and so on) to treat diabetes could reduce glycosylated hemoglobin by 1.67% and was similar to metformin for decreasing glucose and this herb was safer [21, 22]. Jin and colleagues showed that Gegen Qinlian Tang to treat diabetes reduced glucose by 91% [23] and other groups have published data that agree with this finding [24, 25]. Gegen Qinlian Tang is thought to act by reducing glucose metabolism disorders, reducing insulin resistance, increasing tissues sensitivity to insulin, and improving physiological antioxidant functions, as well as being protective for pancreatic function. Research suggests that Gegen Qinlian Tang has similar efficacy to sulfonylureas and biguanides [26, 27].

### 4.2. The Evidence of Single Herb with Bitter Flavor and Cold Property in the Diabetic Treatments

HMs with bitter flavor and cold property are most constituents with alkaloid, about 75% of HMs whose main ingredient is alkaloid. Glycoside also has a high distributive law, about 56% of totality. Alkaloid and glycoside are the main source of bitter flavor and cold property [5].

#### 4.2.1. Rhizoma Coptidis

Rhizoma Coptidis is an isoquinoline alkaloid consisting chiefly of berberine (BBR) [28]. Zhang et al. proved that BBR could reduce glycosylated hemoglobin from 7.5 ± 1.0 to 6.6 ± 0.7% [29]. Others suggest that BBR was 90% effective in reversing diabetes and had few side effects [30]. BBR has the same effect as metformin and rosiglitazone on reducing glucose and glycosylated hemoglobin and is more effective in lipid lowing than them. What is more, BBR can be used as the treatment of DM with liver function impaired lipid lowering [31, 32].

#### 4.2.2. Rhubarb

*Rhubarb* contains emodin, rhein, rhubarb polysaccharide, and so on. Wan's group demonstrated that rhubarb ethanol extracts reduced BMI, glucose, and insulin and regulated lipid metabolism disorders as well as improving insulin resistance in diabetic rats [33]. Zhou et al. confirmed that emodin can prevent increases in fasting blood glucose, lower blood lipids, reduce glycated hemoglobin, and regulate glucose and lipid metabolism [34]. Cheng et al. proved that rhein increased PPAR-*γ* and GluT-4 protein expression in adipose tissue, decreased fasting blood glucose, and improved insulin sensitivity in diabetic rats [35].

#### 4.2.3. *Momordica charantia*

*M. charantia* contains many bioactive constituents, such as saponins, polysaccharide, proteins, and flavonoids [36]. Sun reported that* M. charantia* can reduce glucose and insulin secretion [37]. Song interpreted that* M. charantia* polysaccharide decreased glucose significantly, impacted the secretion of insulin, raised insulin resistance index, and reduced insulin sensitivity index [38]. Total saponins of* M. charantia* could promote hepatic glycogen synthesis, inhibit hepatic glycogen decomposition, and promote insulin sensitivity by increasing peripheral tissue GluT-4 expression [39].

## 5. Mechanisms of Action of HMs with Bitter Flavor and Cold Property in Diabetes

### 5.1. Improving Insulin Sensitivity and Promoting Insulin Secretion

Zhang et al. demonstrated that BBR increased insulin receptor (Ins R) expression both in vitro and in animal models and insulin signaling was significantly enhanced after BBR treatment, confirming BBR as an insulin sensitizer in peripheral tissues via protein kinase C-dependent InsR upregulation. BBR was also safe for patients [31]. According to Zhao et al., after spindle tree wings treatment, compared with control cells, hematoxylin and eosin staining confirmed normal morphology and structure of diabetic pancreatic islet cells, with uneven cytoplasm, clear nuclei, and evenly arranged cells. Staining for insulin expression also revealed statistically significant differences between control and groups treated with spindle tree wings (*P* < 0.01), suggesting that spindle tree wings protected and stimulated islet beta cells while reducing blood glucose, promoting secretion of insulin [40]. Liu used total saponins from balsam pear to intervene T2DM rats, resulting in plasma insulin level being reduced significantly (*P* < 0.01) and insulin sensitivity index being aroused (*P* < 0.05) in the test group compared to the model group. That is to say, balsam pear may improve insulin resistance and promote insulin secretion [41].

### 5.2. Hepatic Regulation of Sugar Metabolism

Yao et al. reported that both hepatic p-Akt and p-GSK-3*β* levels in the liver tissue were upregulated remarkably after geniposide treatment compared to controls (*P* < 0.05). Geniposide can activate downstream phosphorylation of glycogen synthase kinase GSK-3 and inhibit its activity, prevent inhibition of glycogen synthesis, and help to transform downstream glycogen synthase for glucose storage and reducing blood glucose [42]. Liu and coworkers reported that, compared to the diabetic controls, mRNA expression levels of hepatic glucokinase (GcK) in diabetic rats increased and mRNA expression of hepatic glucose-6-phosphatase (G6P) and phosphoenolpyruvate carboxykinase (PEPCK) genes was significantly decreased. Thus, BBR can directly affect GcK, PEPCK, and G6P to improve hepatic glucose utilization in inhibiting glycogen production to lower blood glucose directly [43]. Three different doses of* R. anemarrhenae* polysaccharide were used to treat T2DM. It was found that the content of glycogen in liver was significantly higher than that in control group (*P* < 0.01), indicating that the polysaccharide had a hypoglycemic effect likely via inhibiting decomposition of hepatic glycogen or increasing hepatic glycogen synthesis [44].

### 5.3. Increase the Utilization of Glucose by Peripheral Tissue

Yang et al. proved that euonymus alatus can promote normal adipose cells to take up glucose at low insulin concentrations, suggesting a hypoglycemic mechanism that may be related to promoting insulin resistance and glucose uptake [45]. Song et al. demonstrated that emodin promotes the glucose use by skeletal muscle and hepatic cells by activating AMPK [46]. Zhao et al. reported that emodin improved glucose uptake in adipocytes by upregulating Shirt1 mRNA and protein expression. Compared to control cells, average glucose absorption in emodin cells increased by 46.01% [47]. Kumar et al. suggested that extract of bitter gourd promoted glucose uptake in an L6 muscle model and positively regulated GluT-4, PI3K, and PPAR in glucose transport. Bitter gourd, as a PPAP*γ* activator, can enhance insulin signaling and glucose uptake [48].

### 5.4. Reduced Intestinal Glucose Absorption

*α*-Glucosidase is an intestinal enzyme for digesting carbohydrates and converting them into monosaccharides. Inhibition of this enzyme by drugs suppresses carbohydrate absorption and reduces glucose uptake in the intestine [49]. Chen's group reported that kinetic enzyme assays confirmed that R. anemarrhenae could inhibit *α*-glucosidase. Saponins and flavones were better at inhibiting this enzyme's effect than the antidiabetic drug acarbose. The extracts inhibited *α*-glucosidase 72.9% and 37.91% at 5.6 mg/mL and 0.88 mg/mL, respectively [50]. Liu et al. reported that extracts of mulberry leaves potently inhibited *α*-glucosidase compared to acarbose as a positive control in vitro. Mulberry leaf extract reduced glucose after oral sucrose and starch ingesting in vivo. Mulberry leaf extract decreases blood glucose, and this is related to inhibition of *α*-glucosidase activity [51]. Liu et al. confirmed that no matter in animal experiments or the cell research, BBR can suppress disaccharidase activities and downregulate SI complex mRNA expression in intestinal regions, with beneficial metabolic effects in diabetic states [52].

### 5.5. Others

HMs with bitter flavor and cold property can adjust blood glucose in other ways. Dai's group reported that BBR reduces serum inflammatory modulators in diabetic rats, specifically serum CRP, IL-6, and TNF-*α* were significantly less, and adiponectin significantly increased compared with control group animals (*P* < 0.05) [53]. Zhao and colleagues reported that, in diabetic rats, serum SOD activity decreased and MDA increased significantly. After BBR treatment, MDA decreased and SOD activity was enhanced; thus, BBR may alter glucose metabolism via antioxidant effects and free radical clearance [54]. Ma's laboratory suggested that saponins in* M. charantia* reduce diabetic rat weight and regulate adipose cell secretion which improved insulin resistance and lowered glucose [55].

## 6. Advantages of HMs with Bitter Flavor and Cold Property in the Diabetic Treatment

### 6.1. Multiple Target Points of HMs with Bitter Flavor and Cold Property

#### 6.1.1. Multiple Mechanisms behind Antidiabetic Effect

Different from western drugs which are always having certain one mechanism of lowering glucose, both TCM compound formulas constituted with HMs with bitter flavor and cold property and single herb application have multiple mechanisms in lowering glucose, corelating diabetic state. Huanglian Jiedu Tang reduces glucose by promoting insulin sensitivity and facilitating insulin secretion as well as by inhibiting intestinal disaccharidase activity [56, 57]. More than 5 different mechanisms are attributed to BBR's antidiabetic effects [58], while* M. charantia* has glucose lowering effects as well as pancreatic and extrapancreatic effects [59]. We can conclude that HMs with bitter flavor and cold property have multiple antidiabetic mechanisms, which is more conductive to glucose management for diabetic patients.

#### 6.1.2. Regulating Glucose together with Lipid Metabolism

Obese patients often present with dyslipidemia [8], so treating lipid metabolism disorders would be beneficial. HMs with bitter flavor and cold property just have the effect of regulating the high glucose and lipid state. There was a highly significant decrease in the concentrations of 13 fatty acids following berberine administration in Gu's study [60]. In addition, emodin may improve db/db mouse insulin sensitivity, reduce fasting glucose, and reduce db/db mice serum TG, TC, and LDL-C level. What we can conclude is that emodin can effectively improve human blood lipid metabolism [61, 62]. In Song's study, water extracts from* M. charantia* at the dosage of 10 g/kg could significantly reduce the levels of glucose, TC, and TG, proving that water extracts form* M. charantia* can obviously reduce blood glucose and improve lipid metabolism [63].

### 6.2. Small Toxic/Side Effect

Compared to common gastrointestinal reactions, liver and kidney toxicity and other side effects of toxicity of western drugs, HMs with bitter flavor and cold property have fewer side effects and are more potent, permitting smaller doses to be used. What is more, the toxic/side effects could be avoided by controlling drug dosage or rational compatibility of other HMs. That is why more and more patients prefer to take HMs to control diabetes. In clinical researches, Sun used bitter gourd buccal tablets to intervene diabetes patients for one month and observed no toxicity or allergic reactions. BBR has also been used to treat diabetes and reactive hypoglycemia was not a side effect and only a few patients had gastrointestinal side effects compared with placebo/lifestyle interventions [64]. In addition, rhubarb was reported to lower glucose as well as reducing serum creatinine and protecting renal function, which may delay diabetic-induced kidney disease [65, 66].

## 7. Summary

HMs with bitter flavor and cold property may have multiple antidiabetic mechanisms and have exact antidiabetic effect with small toxic/side effect compared with western drugs. HMs with bitter flavor and cold property can be a complementary and alternative treatment for DM.

In China, Traditional Chinese Medical doctors prefer to apply herbal medicines rather than some compounds or extracts. Along with the internationalization of the Traditional Chinese Medicine, the superiority of TCM treatment of diabetes is more and more obvious and has been recognized by Western doctors. We introduce TCM culture to Western doctors for further knowning about TCM. In the theory of TCM, bitter flavor is an excellent approach to counteract sweet flavor and cold property can clear heat. Taking the core pathogenesis of DM in the early and middle stages—center fullness and internal heat—into consideration, HMs with bitter flavor and cold property can be used as diabetic treatment. Whether in clinical studies, animal researches, cell tests, or the experience of TCM doctors, it is demonstrated that HMs with bitter flavor and cold property have exact antidiabetic effects.

HMs with bitter flavor and cold property are a class of drugs. It is still unclear what their common antidiabetic mechanism is. Maybe some common products of metabolism would exist to lowering blood sugar after HMs with bitter flavor and cold property administration, but this still needs to be further studied.

## References

[1] Xu Y., Wang L., He J. (2013). Prevalence and control of diabetes in Chinese adults. *The Journal of the American Medical Association*.

[2] Xie W. G. (1985). The theory herbal medicine with bitter flavor. *Jiangxi Journal of Traditional Chinese Medicine*.

[3] Chen Q. (1986). The Medicinal theory of herbal medicine with bitter flavor. *Journal of Traditional Chinese Medicine*.

[4] Sun D. D. (1996). Characteristics of herbal medicine with bitter flavor and its agents in a formula. *China Journal of Chinese Materia Medica*.

[5] Lai C. H. S. H. (2015). Analysis of the characteristics of the property and efficacy of drugs with bitter flavor. *Henan Traditional Chinese Medicine*.

[6] Li Y. J., Zhang W., Mayuree T. (2014). The nature and effectiveness of cold bitter medicinals. *Journal of Traditional Chinese Medicine*.

[7] Li G. W. (2003). The importance of prevention and control of type 2 diabetes with rectifing obesity and insulin resistance. *Chinese Journal of Diabetes*.

[8] Wei J.-P., Liu F., Zhou L.-B. (2010). Epidemiological investigation of IGT and Risk factors for DM in Beijing. *Beijing Journal of Traditional Chinese Medicine*.

[9] Tong X.-L., Dong L., Chen L., Zhen Z. (2012). Treatment of diabetes using traditional Chinese medicine: past, present and future. *American Journal of Chinese Medicine*.

[10] Ji H. Y. (2009). The research about origin of splenic pure heat. *Jiangsu Journal of Traditional Chinese Medicine*.

[11] Liu H. Z. (2013). Clinical research of T2DM treated with ‘Pidan’ drug particles. *Guide of China Medicine*.

[12] Zhou L. B. (2008). Discussion of ‘pidan’. *Shanxi Journal of Traditional Chinese Medicine*.

[13] Tong X. L. (2009). The new theory of ‘Pindan’. *China Journal of Traditional Chinese Medicine and Pharmacy*.

[14] Chang B. (2008). The causes and treatment of obesity with T2DM. *Beijing Journal of Traditional Chinese Medicine*.

[15] Lian F. M., Wei Z. X., Lv X. F. (2008). Clinical study on reducing sugar effect of kaiyu qingre-jiangzhuo prescription on T2DM. *World Journal of Integrated Traditional and Western Medicine*.

[16] Zhao Y., Chen L., Dong L., Bi G.-Z., Tong X.-L. (2013). Influence of Kaiyu Qingre Fang on *β*-cell function in patients with obese type 2 diabetes (syndrome of heat depression in liver and stomach). *Journal of Beijing University of Traditional Chinese Medicine*.

[17] Piao C.-L., Tong X.-L., Han X. (2011). The experiment study of Kaiyuqingre's prescription on the expression of sterol regulatory element binding protein-1c and fatty acid synthase in peritoneal adipose tissue of spontaneous type 2 diabetes mellitus rats(OLETF rats). *Chinese Archives of Traditional Chinese Medicine*.

[18] Zhen Z. (2009). Influence of Kaiyuqingre formula on glycolipin metabolism in spontaneous obese T2DM rats. *Chinese Archives of Traditional Chinese Medicine*.

[19] Tong X. L., Wu S. T., Lian F. M. (2013). The safety and effectiveness of TM81, a Chinese herbal medicine, in the treatment of type 2 diabetes: a randomized double-blind placebo-controlled trial. *Diabetes, Obesity and Metabolism*.

[20] Song J. (2010). Effect of Tang Min Ling pill on PPAR*α* and insulin secretion. *Chinese Journal of Basic Medicine in Traditional Chinese Medicine*.

[21] Lian F. M., Tong X. L., Bai Y. (2009). Analysis on the effect of Qingre Jiangzhuo prescription on overweight subjects of type 2 diabetes mellitus patients. *Chinese Journal of Information on Traditional Chinese Medicine*.

[22] Zhu Y. (2010). Effect of Qingre-Jiangzhuo prescription on islet *β* cell function of type 2 diabetes mellitus combined metabolic syndrome patients. *Chinese Journal of Information on Traditional Chinese Medicine*.

[23] Jin L. (2012). Treatment of 120 diabete patients with Gegenqinlian prescription. *Journal of Changchun University of Traditional Chinese Medicine*.

[24] Wang F. (2005). The experience of Professor Zhang treat DM with Gegenqinlian prescription. *Journal of Traditional Chinese Medicine*.

[25] Zhao L. H. (2011). Clinial examples of treatment for type 2 diabetes by Professor Tong Xiao-lin using Gegen Qinlian decoction. *Chinese Journal of Experimental Traditional Medical Formulae*.

[26] Pan J. Q., Han C., Liu H. (2000). Experimental study on hypoglycemic effects of gegenqinliangtang. *Chinese Journal of New Drugs*.

[27] Li Y.-M., Fan X.-M., Wang Y.-M., Liang Q.-L., Luo G.-A. (2013). Therapeutic effects of Gegen Qinlian decoction and its mechanism of action on type 2 diabetic rats. *Acta Pharmaceutica Sinica*.

[28] Wang Y. (2014). Modern research progress of huanglian. *China Journal of Chinese Medicine*.

[29] Zhang Y., Li X., Zou D. (2008). Treatment of type 2 diabetes and dyslipidemia with the natural plant alkaloid berberine. *The Journal of Clinical Endocrinology & Metabolism*.

[30] Mi M. (2015). Curative effect analysis about T2DM treated with berberine. *Diabetes New World*.

[31] Zhang H., Wei J., Xue R. (2010). Berberine lowers blood glucose in type 2 diabetes mellitus patients through increasing insulin receptor expression. *Metabolism: Clinical and Experimental*.

[32] Zhao W., Xue R., Zhou Z.-X., Kong W.-J., Jiang J.-D. (2008). Reduction of blood lipid by berberine in hyperlipidemic patients with chronic hepatitis or liver cirrhosis. *Biomedicine and Pharmacotherapy*.

[33] Wang S. F. (2007). The experimental study of the effect of alcohol extract from *Rheum palmatum* L on diabetes type 2 rats with insulin resistance. *Modern Journal of Integrated Traditional Chinese and Western Medicine*.

[34] Zhou L.-E., Xu Y.-Y., Chen W.-B., Zeng W.-J., Wang Y.-M., Yong O.-Y. (2012). The experimental study of Emodin improve glucolipid metabolism of mice with type 2 diabetes. *Journal of Chinese Medicinal Materials*.

[35] Cheng C., Miao J., Yoshiaki M. (2008). Rhein improves insulin sensitivity of diabetic rats by increasing the protein expression levels of PPAR-*γ* and GluT-4 in adipose tissue. *Chinese Journal of Diabetes*.

[36] Chen Z. H. (2014). Advance on hypoglycemic function of bitter gourd components. *Journal of Food Industry*.

[37] Sun F. Q., Zhang G., Huang B., Bai J., Yu X. (2000). Clinical observation of using Bitter melon to treat DM. *Liaoning Journal of Practical Diabetology*.

[38] Song J. P. (2012). The influence of blood sugar and insulin levels of Balsam pear polysaccharide in mice with diabetes. *China Practical Medicine*.

[39] Ma C.-Y., Yu H.-Y., Wang H.-J., Geng L.-J., Guan H.-Q. (2014). Hypoglycemic mechanism of total saponins of *Momordica charantia* in type 2 diabetes mellitus rats. *Tianjin Medical Journal*.

[40] Zhao M. M., Xie M. Z., Li L. D., He J. F., Wang Y. (2010). Effect of *Euonymus alatus* on islet *β*-cellin type 2 diabetic rats. *Journal of Traditional Chinese Medicine University of Hunan*.

[41] Liu Y. S. (2010). Effects of total saponins from balsam pear on insulin resistance, adiponectin and leptin in rat model of type 2 diabetes. *Chinese Journal of Experimental Traditional Medical Formulae*.

[42] Yao D.-D., Shu L., Yang L., Jia X.-B., Sun M. (2014). Hypoglycemic effect of geniposide and its relative mechanism. *Chinese Traditional and Herbal Drugs*.

[43] Liu X. H. (2008). Effect of berberine on mRNA expression levels of hepatic glucokinase, glucose-6-phosphatase and phosphoenolpyruvate carboxykinase in type 2 diabetic Chinese hamsters. *Chinese Journal of Comparative Medicine*.

[44] Lu S. H., Sun H. W., Wang J. Y., Wei X. B., Xu H. Y. (2003). Study on the hypoglycemic effect and mechanism of *Rhizoma anemarrhenae* polysaccharide. *Chinese Journal of Biochemical Pharmaceutics*.

[45] Yang H.-Y., Wang Q.-J., Zhu D.-N., Zhao X.-C. (2004). Effect of extracts from *Euonymus alatus* Sieb. on peripheral glucose uptake of adipocytes. *Chinese Journal of Natural Medicines*.

[46] Song P., Kim J. H., Ghim J. (2013). Emodin regulates glucose utilization by activating amp-activated protein kinase. *The Journal of Biological Chemistry*.

[47] Liu Y.-H., Teng J.-Y., Zheng Z.-X. (2014). Emodin regulates glucose uptake by activating sirtl in 3T3-L1 adipocytes. *Journal of Nanjing University of Chinese Medicine*.

[48] Kumar R., Balaji S., Uma T. S., Sehgal P. K. (2009). Fruit extracts of Momordica charantia potentiate glucose uptake and up-regulate Glut-4, PPAR*γ* and PI3K. *Journal of Ethnopharmacology*.

[49] Yin J., Zhang H., Ye J. (2008). Traditional Chinese medicine in treatment of metabolic syndrome. *Endocrine, Metabolic and Immune Disorders—Drug Targets*.

[50] Chen L. H., Pan Z. H., Cao Y. L., Ma Q. Y. (2013). A screen of *α*-glucosidase inhibitors from rhizoma anemarrhenae. *Food & Machinery*.

[51] Liu Y.-H., Teng J.-Y., Zheng Z.-X. (2004). Effect of mulberry leaves extract on postprandial blood sugar and activity of alpha-glucosidase. *Chinese Journal of Clinical Rehabilitation*.

[52] Liu L., Yu Y.-L., Yang J.-S. (2010). Berberine suppresses intestinal disaccharidases with beneficial metabolic effects in diabetic states, evidences from in vivo and in vitro study. *Naunyn-Schmiedeberg's Archives of Pharmacology*.

[53] Dai J. F. (2014). Impact of berberine, CRP, IL-6, TNF-*α* and adiponectinon type 2 diabeie rats in blood glueose. *Chinese Journal of Trauma and Disability Medicine*.

[54] Zhao H. T. (2010). The effect of berberine on SOD and MDA in the serum ofdiabetes rats. *Journal of Mudanjiang Medical College*.

[55] Ma C. Y., Yu H. Y., Wang H. J., Guan H. Q. (2013). Effects of total saponins of momordica charantia on plasma lipid and adipocyte factors in type 2 diabetes rats. *Pharmacology and Clinics of Chinese Materia Medica*.

[56] Leng S. H., Lu F. E., Tu Q. N., Xu L. J., Yang M. W., Wang K. F. (2003). Effects of Huang Lian Jie Du decoction on blood glucoseand lipids metabolisms in type II diabetic rats. *Chinese Journal of Basic Medicine in Traditional Chinese Medicine*.

[57] Deng Y.-X., Liu X.-D., Liu L., Chen Y.-S. (2010). Effects of huanglian jiedu decoction on activities of intestinal disaccharidases in diabetic rats. *Chinese Traditional and Herbal Drugs*.

[58] Pang B., Zhao L., Zhou Q. (2015). Application of berberine on treating type 2 diabetes mellitus. *International Journal of Endocrinology*.

[59] Chaturvedi P. (2012). Antidiabetic potentials of momordica charantia: multiple mechanisms behind the effects. *Journal of Medicinal Food*.

[60] Gu Y., Zhang Y., Shi X. (2010). Effect of traditional Chinese medicine berberine on type 2 diabetes based on comprehensive metabonomics. *Talanta*.

[61] Zhu H.-Q., Liang L.-M., Wang P.-J., Chen G. (2011). Effect of emodin on blood glucose and lipid in mice model with type 2 diabetes. *Progress in Modern Biomedicine*.

[62] Zhang W. J. (2014). Effect of *Rhubarb* on the contents of serum TC, TG and HDL-C in hyperlipidemia model rats. *Journal of Gansu College of Traditional Chinese Medicine*.

[63] Song C. W., Zhou Z., Peng M. (2011). Effects of water extract from *Momordica charantia* L. on serum glucose and lipids of experimental diabetic mice. *China Pharmacist*.

[64] Narenqimuge, Zhao T.-Y., He M., Tian C. (2012). Effectiveness and safety of berberine in the treatment of type 2 diabetes: a systematic review. *Chinese Journal of Evidence-Based Medicine*.

[65] Dai C.-S., Liu Z.-H., Chen H.-P. (1999). The long-term experimental study of STZ induced diabetic rats by rhubarb. *Chinese Journal of Nephrology Dialysis & Transplantation*.

[66] Hu J., Miao J., Duan S.-F., Wang Y.-L., Li N.-J., Zou Y.-T. (2014). Effects of rhein on urinary protein excretion in type2 diabetic model rats. *Chinese Pharmacy*.

